# Differences in Redox Biomarkers in Bronchoalveolar Lavage Fluid of Leisure Horses With and Without Severe Equine Asthma: Preliminary Results

**DOI:** 10.3390/ani16060882

**Published:** 2026-03-12

**Authors:** Francesca Bindi, Valentina Vitale, Dania Cingottini, Anna Pasquini, Mariangela Longini, Giulia Tagliaferri, Francesca Bonelli, Irene Nocera, Micaela Sgorbini

**Affiliations:** 1 Department of Veterinary Sciences, University of Pisa, 56124 Pisa, Italy; francesca.bindi@phd.unipi.it (F.B.); anna.pasquini@unipi.it (A.P.); francesca.bonelli@unipi.it (F.B.); irene.nocera@unipi.it (I.N.); micaela.sgorbini@unipi.it (M.S.); 2 Department of Animal Medicine and Surgery, Universidad CEU-Cardenal Herrera, CEU Universities, 46115 Alfara del Patriarca, Valencia, Spain; valentina.vitale@uchceu.es; 3 Department of Molecular and Developmental Medicine (DMMS), University of Siena, 53100 Siena, Italy; mariangela.longini@unisi.it; 4 RW Equine Vet Ltd., Egmont Farm, Coppards Lane, Northiam, Rye TN31 6QN, East Sussex, UK

**Keywords:** severe equine asthma, oxidative stress, antioxidant capacity, non-protein-bound iron, advanced oxidation protein products, reactive oxygen metabolites

## Abstract

Equine asthma is a common chronic respiratory disease characterized by airway inflammation and oxidative imbalance, particularly in its severe form. The aim of this study was to evaluate pro-oxidative substances, oxidative products, and antioxidant defense biomarkers in bronchoalveolar lavage fluid (BALF) from horses with and without a history of severe equine asthma (SEA). This prospective observational study included 21 leisure horses classified as SEA or non-SEA based on clinical history and BALF cytology. Bronchoalveolar lavage fluid was analyzed for derivatives of reactive oxygen metabolites (d-ROMs), non-protein-bound iron (NPBI), advanced oxidation protein products (AOPP), and biological antioxidant potential (BAP) using validated analytical methods. Horses with SEA showed significantly higher NPBI concentrations compared with non-SEA horses, whereas no significant differences were observed for BAP level, AOPP and d-ROMs concentrations between groups. This finding indicates an altered local redox balance in the airways of horses with severe equine asthma, characterized by increased pro-oxidative potential. Overall, the results support the involvement of oxidative mechanisms in the pathophysiology of severe equine asthma and highlight the potential value of bronchoalveolar lavage fluid biomarkers for improving disease characterization.

## Simple Summary

Equine asthma is a common long-term respiratory disease in horses that causes breathing difficulty and airway inflammation, particularly in its severe form. Inflammation in the lungs can lead to an imbalance between harmful molecules, known as oxidants, and the body’s natural defenses, called antioxidants. This imbalance may contribute to lung damage and the progression of the disease. The aim of this study was to evaluate markers of oxidative damage and antioxidant protection in fluid collected from the lungs of horses with and without severe equine asthma. We found that horses with severe equine asthma had higher levels of a reactive form of iron that can promote oxidative damage. Other markers showed no clear differences between groups. These results indicate that a pro-oxidative status is present locally in the lungs of affected horses. A better understanding of these processes may help improve disease monitoring and support the development of management strategies, such as nutritional or environmental interventions, to improve respiratory health and welfare in horses.

## 1. Introduction

Equine asthma (EA) is a common condition among horses and includes two main phenotypes: severe equine asthma (SEA) and mild-to-moderate equine asthma (MEA). Severe equine asthma is prevalent in horses older than 7 years. It is characterized by exercise intolerance and marked by increased respiratory effort at rest, with a bronchoalveolar lavage (BAL) fluid neutrophil proportion greater than 25%. Mild to moderate equine asthma (MEA) is a pauci-symptomatic disease, usually affecting young to middle-aged horses. It is typically associated with poor performance and excessive mucus accumulation in the tracheobronchial tree. In some cases, a chronic cough lasting longer than three weeks may be observed. Mild to moderate equine asthma can be further classified into subtypes based on the predominant inflammatory cell populations identified in bronchoalveolar lavage fluid (BALF). Specifically, a mild increase in neutrophils (>10%), eosinophils (5%), and mastocytic cells (>5%) may be observed. A mixed inflammatory subtype is recognized when at least two of these cells are concurrently increased [1].

Following antigen exposure in horses with SEA, circulating neutrophils are rapidly activated and release chemokines that attract inflammatory cells into the airway lumen within hours. This neutrophil-dominated inflammatory response plays a central role in disease pathogenesis and persistence [2]. A comparable process occurs in human asthma, where neutrophils secrete pro-inflammatory cytokines that enhance further neutrophil recruitment, airway inflammation, and the generation of reactive oxygen species (ROS) [3]. Persistent neutrophil activation within the airway lumen results in excessive oxidative stress characterized by an increase in reactive oxygen species (ROS) released from inflammatory cells. In studies performed in human medicine, the levels of total antioxidants were inversely related to the severity of asthma, and patients with acute asthmatic attacks showed increased oxidative stress biomarkers [4,5]. The resulting imbalance between ROS generation and antioxidant defenses promotes oxidative damage to airway tissues, influencing the severity of the disease and treatment [5,6].

Derivatives of reactive oxygen metabolites (d-ROMs) have been used to assess oxidative stress status in patients with asthma, as they reflect early-stage ROS-induced damage before degradation into more stable end products. Studies in patients with bronchial asthma have demonstrated that serum d-ROMs levels are higher than those observed in non-asthmatic individuals and that they correlate with disease severity and the degree of airflow limitation [7,8]. Along with d-ROMs, non-protein-bound iron (NPBI) has been considered as a marker in asthmatic patients since this form of iron, not bound to transferrin, can generate free reactive radicals. Thus, as a pro-oxidative compound, NPBI can contribute to oxidative stress and inflammation in the airways [9,10,11,12].

Advanced oxidation protein products (AOPP) are terminal markers of protein oxidation induced by free radicals. In human patients with asthma, increased plasma AOPP levels have been reported and are thought to contribute to disease pathogenesis by promoting inflammation and airway hyperreactivity. Advanced oxidative protein products (AOPP) concentration has been found to increase in asthmatic patients affected by uncontrolled asthma. This finding supports the hypothesis that conditions of uncontrolled asthma may strongly evoke oxidative stress [13].

Biological antioxidant potential (BAP) measures the overall antioxidant capacity in blood. In human patients’ asthma, particularly severe cases, studies have linked elevated oxidative stress to reduced BAP levels, contributing to airway inflammation and remodeling [8].

In human asthma, elevated oxidative stress leads to depletion of antioxidants and dysfunction of antioxidant enzymes, which in turn promotes increased mucus secretion, alterations in capillary endothelial integrity, and leakage of ROS into the peripheral circulation [14,15].

Six studies [16,17,18,19,20,21] are present in the literature concerning oxidative stress biomarkers and respiratory diseases, but the differences in sampling sites, whether from the lungs or the systemic circulation, as well as the wide range of oxidative stress and antioxidant biomarkers analyzed, complicate direct comparisons among studies. The selection of appropriate biomarkers and the compartment in which they are measured is therefore critical for accurately capturing redox imbalance in airway disease, and for translating findings into clinically useful diagnostic or monitoring tools [4,21].

This study aimed to measure pro-oxidative substances (d-ROMS and NPBI), oxidative products (AOPP), and antioxidant defense biomarkers (BAP) in bronchoalveolar lavage fluid (BALF) in a cohort of leisure horses.

## 2. Materials and Methods

### 2.1. Animals

This prospective, observational, in vivo clinical study was approved by the Ethical Committee of the University of Pisa (n. 53/2020).

A cohort of 21 horses with and without a history of SEA was enrolled. All horses underwent a complete clinical examination, thoracic ultrasound assessment, and BAL, which were performed for clinical purposes.

Based on clinical history and cytological findings [1], horses were divided into non-SEA and SEA groups.

### 2.2. Thoracic Ultrasound and BAL Procedure

Thoracic ultrasound assessment was performed on non-sedated, manually restrained horses using a portable machine (MyLab SigmaVET, Esaote, Firenze, Italy) and multifrequency probe [22].

Bronchoalveolar lavage (BAL) was performed after at least 8 h of fasting and under sedation with alfa2-agonist (detomidine, 0.01 mg/kg IV; Dorum^®^, ACME S.r.l., Cavriago (RE), Italy), as previously reported [23]. Horses were restrained in a stock, and a nose twitch was applied. Briefly, BAL was performed using a commercially available equine BAL catheter, 3 m in length and 11 mm in outer diameter, equipped with an inflatable cuff (Bivona Inc., Gary, IN, USA). The tube was introduced blindly through the ventral meatus into the pharynx. The head of the horse was then stretched into a horizontal position relative to the ground, and the tube was advanced caudally into the trachea, with the head maintained in an extended position. To reduce tracheal sensitivity and suppress coughing during catheter advancement, lidocaine (Lidocaina 2%, Ecuphar Italia S.r.l., Milano, Italy) diluted to a final concentration of 0.66% in sterile 0.9% saline (NaCl 0.9%, B.Braun Milano S.p.A., Milano, Italy) was administered. The BAL catheter was advanced at a moderate pace until gentle resistance was perceived. The cuff was then inflated with air, and the tube was secured in position by pressing it against the nasal septum. At this point, the BAL catheter was positioned in the third- and fourth-generation bronchi to sample a broad lung area and minimize variability in differential cell count evaluation. Subsequently, 300 mL of warm, sterile, isotonic crystalloid solution (NaCl 0.9%, B.Braun Milano S.p.A., Milano, Italy) was instilled through the tube. Bronchoalveolar lavage fluid was recovered by manual aspiration using 60 mL syringes, with a minimum recovery of 40% of the infused volume. The cuff was then deflated, and the tube was removed.

One aliquot of BALF was collected in ethylenediaminetetraacetic acid (EDTA) tubes to reduce cell clumping and processed within 1 h from the collection for cytological analysis, and 4 aliquots were collected in heparinized tubes and stored at −80 °C.

### 2.3. Cytological Analysis

The BALF total nucleated cell count (TNCC) was determined on EDTA samples using an automated counter (Hecovet, SEAC-RADIM Co, Firenze, Italy). Slide preparation was conducted by cytocentrifugation (Statspin Cytofuge 2, Beckman Coulter Inc., Brea, CA, USA) with 400 μL per slide and 300 rpm for 10 min. Air-fixed slides were stained with May-Grundwald Giemsa using an automatic colorimeter machine (Aerospray Hematology Slide Stainer mod. 7150, Delcon Srl, Grassobbio (BG), Italy) for a duration of 12 min. The stained smears were scanned at low magnification (×200), then at higher magnification (×400) for optimal area coverage. Cells were recognized and counted using oil immersion (×1000). The slide was examined using a systematic scanning technique, starting from the upper edges and progressing toward the center and lower edges to avoid repeated evaluation of the same areas. For each smear, 400 cells were counted for differential cell analysis using a bright-field optical microscope.

### 2.4. Oxidative Stress and Antioxidant System Parameters

All BALF samples were evaluated for d-ROMs, NPBI, AOPP, and BAP.

d-ROMs concentration was assessed using a spectrophotometric method, following the manufacturer’s instructions (Diacron Srl, Grosseto, Italy). The test measures the concentration of hydroperoxides, a class of chemical oxidant species belonging to the ROM group. In brief, in the d-ROMs, reactive oxygen metabolites, in the presence of iron, can generate alkoxyl and peroxyl radicals, according to the Fenton reaction. Such radicals can then oxidize an amine (N,N-dietylparaphenylendiamine), thus producing a pink-colored derivative, which is photometrically quantified at 505 nm. The d-ROMs concentration is directly proportional to the color intensity and is expressed as Carratelli Units (1 CARR U = 0.08 mg hydrogen peroxide/dL) [24].

BAP concentration was measured using a spectrophotometric method, following the manufacturer’s instructions (Diacron srl, Grosseto, Italy). The test measures BAP as the capacity of the BALF sample to reduce iron from ferric (Fe^3+^) to ferrous form (Fe^2+^). In the BAP test, the addition of a BALF sample to a colored solution, obtained by mixing a ferric chloride solution with a thiocyanate derivative solution, causes a discoloration, whose intensity is measured photometrically at 505 nm and is proportional to the ability of the BALF to reduce ferric ions. The results are expressed as mmol/L of reduced ferric ions [25]. The stability of the d-ROMs and BAP assays in stored samples has been evaluated in previous studies [26], and the concentrations in BALF have also been previously reported [27].

AOPP concentration was measured as previously described using a spectrophotometric assay. The AOPP was calibrated with chloramine-T solutions that absorb at 340 nm in the presence of potassium iodide. The AOPP concentration was expressed as μmol/L chloramine-T equivalents [28].

The NPBI concentration was measured by high-performance liquid chromatography (HPLC) using the method previously described [29]. The method is based on the preferential binding of NPBI to disodium nitrilotriacetic acid (NTA). The NPBI concentration was expressed as μmol/L.

### 2.5. Statistical Analysis

Data normality was assessed using the Shapiro–Wilk test. Unadjusted group comparisons were initially performed using Mann–Whitney tests. To account for the potential confounding effect of age, multiple linear regression models were performed for each biomarker, including the group as a fixed factor and age as a continuous covariate. The interaction term (group × age) was initially tested and removed from the final models when not significant. Regression assumptions were evaluated by visual inspection of residual plots. To control for multiple comparisons across the four biomarkers, Holm’s step-down procedure was applied. All tests were two-tailed, and statistical significance was set at *p* < 0.05. Statistical analyses were performed using JMP software Pro 18 (SAS Institute Inc., Cary, NC, USA).

## 3. Results

One of the 21 horses enrolled was excluded due to non-diagnostic BALF cytology. Of the 20 horses, 14 out of 20 were assigned to the non-SEA group, and 6 of 20 were assigned to the SEA group. The median age was 9 (5–15) years old and 14.5 (13–20) years old for the non-SEA and SEA groups, respectively. The non-SEA group consisted of 13 females and 1 gelding; the median body weight was 520 kg. The SEA group consisted of 5 females and 1 stallion; the median body weight was 550 kg. All horses included in the study were Standardbreds.

All the SEA horses were in remission: no nasal discharge, respiratory effort, or cough were present, respiratory rate was within reference intervals (<20 bpm), and no wheezes or increased respiratory murmur were present in all the SEA horses. All horses were housed in a paddock 24 h/day with hay administered in feeders. No treatment with corticosteroid and/or bronchodilators was performed in the last 30 days.

Results of BALF cytology have been reported in Table 1; results of d-ROMs, NPBI, AOPP, and BAP tests in the non-SEA and SEA groups have been reported in Table 2.

Multiple linear regression analyses, including group and age, were performed to adjust for age differences between groups. The group × age interaction was not significant for any biomarker and was therefore removed from the final models. After adjustment for age, only NBPI remained significantly associated with the group (*p* = 0.0041), whereas d-ROMs, AOPP, and BAP were not significant. Age showed a borderline association with d-ROMs and NBPI, but was not significantly associated with AOPP or BAP.

After Holm correction for multiple comparisons across the four biomarkers, only NBPI remained significantly associated with the group (adjusted *p* = 0.0164).

## 4. Discussion

The present study evaluated pro-oxidative substances, oxidative products, and antioxidant defense biomarkers in BALF in a cohort of leisure horses with and without a clinical history of SEA. Overall, differences in oxidative stress and antioxidant biomarkers have been found between the two groups of horses included in the present study.

Significantly, a higher concentration of NPBI was measured in the SEA group compared to the non-SEA group. Non-protein-bound iron (NPBI) refers to circulating iron that is not bound to transferrin, ferritin, or heme and is instead associated with low-molecular-weight ligands. This iron pool is redox-active and potentially toxic, as it can promote oxidative stress and pathological iron deposition in tissues [30]. An alteration in iron homeostasis characterized by local iron overload has been reported in BALF from human patients with asthma and chronic obstructive pulmonary disease (COPD) [31,32,33]. In horses with SEA, oxidative stress is a well-established feature, particularly during disease exacerbations [18,34,35]. The NPBI has not been previously characterized in horses with SEA. However, iron is mechanistically relevant in oxidative stress settings, as neutrophil-derived hydrogen peroxide can react with redox-active iron to generate highly reactive hydroxyl radicals, thereby amplifying oxidative tissue injury, as already reported [9,10,11,30].

No differences in BAP concentration were measured between the two groups. This parameter represents an index of the overall reducing power of circulating antioxidants, providing an integrated measure of the capacity to counteract oxidative stress. Previous studies in human medicine reported a significant reduction in the antioxidant index and BAP in patients with COPD compared with healthy controls, and a significant decrease in the BAP during COPD exacerbation because of oxidative stress [36,37,38,39,40]. This reduction may reflect an imbalance between oxidant production and antioxidant defenses associated with chronic airway inflammation, driven by neutrophil-derived reactive oxygen species [41]. In horses, BAP has been investigated as an index of antioxidant activity in athletic horses and neonatal foals [42,43,44]. To date, no studies have evaluated BAP in relation to EA; however, the lack of differences between the two groups regarding BAP concentration might support the hypothesis that the antioxidant capacity within the alveolar microenvironment is similar between SEA vs. non-SEA horses. These findings are not in line with previous studies reporting redox alterations and antioxidant depletion in BALF from horses with SEA. The difference might be due to the low number of horses included in the SEA group. 

The concentration of AOPP was similar in horses with SEA compared with the non-SEA group. AOPP has been suggested to facilitate the removal of oxidized albumin from the alveolar lining fluid, thereby limiting local reactive oxygen species accumulation [45]. AOPP concentration was assessed in preterm newborns and found to be higher in those affected by neonatal respiratory distress syndrome compared to those not affected [46]. AOPP concentration was also evaluated in BALF of children affected by Mycoplasma pneumoniae pneumonia and found to be higher compared to healthy children [47]. AOPP concentration was found to be higher in the blood of men affected by COPD in exacerbation [38]. The results found are not in line with the literature in human medicine. Since the horses included in the SEA group were in remission, the differences might be due to a mild oxidative injury compared to exacerbated COPD or pneumonia.

No significant differences were detected between horses with SEA and those in the non-SEA group with respect to d-ROM concentrations in BALF. In human patients with severe asthma and COPD, higher concentrations of d-ROMs have been reported, together with a positive correlation with central airway resistance values, indicating that oxidative stress is associated with altered pulmonary mechanics [7,8]. To our knowledge, data on d-ROMs in BALF from horses with EA are currently lacking. This finding may indicate that hydroperoxide-related oxidative markers in BALF are relatively stable across disease status, or that local antioxidant buffering limits measurable differences.

A significant difference in age was found between the two groups of horses included in this study. In particular, horses in the SEA group were older compared with those in the non-SEA group. This result was expected, as SEA usually affects horses older than 7 years. Biomarkers are significantly influenced by age, as aging is characterized by increased accumulation of reactive oxygen and nitrogen species (RONS) and compromised antioxidant defenses. Markers of oxidative stress tend to increase with age because age-related mitochondrial dysfunction leads to enhanced ROS production, resulting in higher levels of oxidized DNA, lipids, and proteins. On the other hand, antioxidant defenses decline; the body’s natural antioxidant defense system becomes less efficient with advancing age, resulting in higher net oxidative stress [48]. In this study, only NBPI concentration was found to be different between the two groups. The difference in age between the two groups included in this study might have partially influenced the statistical difference between the SEA vs. non-SEA horses found for NBPI. 

Given the pivotal role of oxidative stress in the pathogenesis of EA, antioxidant supplementation may offer therapeutic benefits. Several studies have demonstrated that antioxidant compounds, such as *Allium sativum* [49,50], *Glycyrrhiza glabra* [51], *Arthrospira platensis* [52], and omega-3 and omega-6 fatty acids [53] can mitigate inflammation, reduce ROS production, and limit mucus accumulation. *Allium sativum* is claimed to have numerous beneficial properties, and it is commonly used for the treatment of respiratory diseases and infections in horses’ lungs. The garlic activity is related to the S-Allyl cysteine, an active component of *Allium sativum*. This active component showed various pharmacological effects, such as anti-inflammatory and antioxidant activities. Garlic supplementation has shown a suppressive effect on inflammation in allergic airways in mice [49]. Moreover, garlic supplementation in horses seemed to reduce the tracheal symptoms and accumulation of tracheal exudates. Additionally, neutrophils in tracheal mucous were lower in the treated group compared to controls [50]. *Glycyrrhiza glabra*, also known as licorice, showed potent anti-inflammatory effects, reducing pro-inflammatory cytokines and scavenging ROS. The two main active components of licorice include glycyrrhizin and glycyrrhitinic acid. *Glycyrrhiza glabra* seemed to alleviate oxidative stress and inflammation in COPD rats [51]. In horses, the effect of a nutraceutical supplement containing *Glycyrrhiza glabra* has been studied in racehorses [54]. Alpha-linolenic acid and linolenic acid are essential polyunsaturated fatty acids (PUFAs) and precursors of the omega-3 and omega-6 series of PUFAs, respectively. The role of omega-3 PUFAs in inflammation is the decrease in the generation of inflammatory cytokines, such as tumor necrosis factor alpha, interleukin (IL) 1 beta, IL-6, IL-8, and reduced expression of adhesion molecules. The beneficial effect of omega-3 PUFAs has been studied in people with chronic pulmonary inflammatory conditions. Horses affected by SEA and supplemented with omega-3 and omega-6 showed a greater reduction in total leukocyte count in BALF [53]. Antioxidant supplementation, only when also combined with environmental management, has been associated with improved respiratory clinical signs and decreased mucus accumulation [53,54]. The management consists of reducing the exposure to the respirable dust by decreasing dust in hay and bedding (i.e., replacing hay feed and straw bedding with wood bedding and a complete pelleted diet or haylage, using mechanical ventilation in stables) [1]. Such non-pharmacological interventions are of particular interest since pharmacological treatments are restricted during competition [54].

This study has some limitations that should be acknowledged. Firstly, the relatively small sample size, particularly in the SEA group, may have limited the statistical power to detect differences for d-ROMs, BAP, and AOPP. Further studies would be necessary to improve the statistical power of the results. Secondly, for convenience, the study population consisted of leisure horses and was cytologically classified into SEA and non-SEA groups; therefore, the non-SEA group may have included both healthy horses and horses clinically affected by MEA, potentially attenuating differences between the two groups. Since the horses included did not undergo athletic exercise, we could not have assessed MEA-related symptoms, such as poor performance. Thirdly, the disease phase (exacerbation or remission) at the time of sampling was not standardized and may have influenced oxidative stress and antioxidant biomarker concentrations. Moreover, the cross-sectional design does not allow the determination of causal relationships between oxidative imbalance and SEA pathophysiology. Lastly, the two groups included in this study showed differences in age. Biomarkers are significantly influenced by age, as aging is characterized by increased ROS accumulation and reduced antioxidant defenses, resulting in higher net oxidative stress [46]. In human patients, the relationship between the onset of disease—such as Chronic Obstructive Pulmonary Disease (COPD), Acute Respiratory Distress Syndrome (ARDS), or Idiopathic Pulmonary Fibrosis (IPF)—age, and oxidative stress has been studied [55]. Thus, the differences in NPBI found between the two groups of horses might have been influenced by the older age of the SEA horses compared with the non-SEA ones. Considering the above, caution is warranted when interpreting the results.

## 5. Conclusions

This study provides evidence of a pro-oxidative shift in the BALF of horses with SEA, as indicated by higher NPBI concentrations. No significant differences in BAP, AOPP, or d-ROM concentrations were observed between SEA and non-SEA horses. These findings may suggest that oxidative balance within the alveolar microenvironment remains relatively stable in horses with SEA during remission. Overall, these findings highlight the complexity of oxidative processes in EA and support further investigation of oxidative and antioxidant biomarkers as potential tools for improving disease characterization and management.

## Figures and Tables

**Table 1:** Total nucleated cell count (TNCC) expressed as cells/μL (reference interval: <530 cells/cells/μL), and macrophages (M), lymphocytes (L), neutrophils (N), mast cells (MC), and eosinophils (E) expressed as percentages in horses without severe equine asthma (non-SEA group) and horses with severe equine asthma (SEA group) included in the study.

**Non-SEA Group**						
	**TNCC**	**M**	**L**	**N**	**MC**	**E**
1	400	36	56	7	0	1
2	400	42	50	6	1	0
3	430	30	63	4	0	3
4	380	34	54	8	1	3
5	450	28	65	7	0	0
6	420	32	64	4	0	0
7	380	34	60	6	0	0
8	470	39	56	5	0	0
9	500	30	60	8	2	0
10	400	38	48	8	0	5
11	390	44	48	7	0	1
12	380	44	48	7	0	1
13	300	55	41	4	0	0
14	400	65	26	9	0	0
**SEA Group**						
	**TNCC**	**M**	**L**	**N**	**MC**	**E**
1	100	51	23	26	0	0
2	200	40	26	25	3	6
3	200	29	36	34	1	0
4	200	35	36	28	1	0
5	200	25	28	46	0	1
6	400	45	41	14	0	0

**Table 2:** Derivatives of reactive oxygen metabolites (d-ROMs), non-protein-bound iron (NPBI), advanced oxidation protein products (AOPP), and biological antioxidant potential (BAP) were measured in bronchoalveolar lavage fluid from horses cytologically without severe equine asthma (non-SEA) and horses with severe equine asthma (SEA).

Group	Non-SEA (*n* = 14)	SEA (*n* = 6)	*p* Value
d-ROMs (CARR U) ± SE	193.51 ± 18.51	234.67 ± 33.52	0.3646
NPBI (μmol/L) ± SE	2.82 ± 0.90	9.94 ± 1.63	0.0041 *
AOPP (μmol/L) ± SE	2.72 ± 0.71	2.09 ± 1.28	0.7149
BAP (mmol/L) ± SE	3.39 ± 0.57	1.07 ± 1.03	0.1044

Abbreviations: d-ROMs—derivatives of reactive oxygen metabolites; NPBI—non-protein-bound iron; AOPP—advanced oxidation protein products; BAP—biological antioxidant potential; SE—standard error; SEA—severe equine asthma; non-SEA—horses without severe equine asthma. * statistically different.

## Data Availability

The original contributions presented in this study are included in the article. Further inquiries can be directed to the corresponding author.

## Abbreviations

The following abbreviations are used in this manuscript:

EA	Equine asthma
SEA	Severe equine asthma
BAL	Bronchoalveolar lavage
MEA	Moderate equine asthma
ROS	Reactive oxygen species
d-ROMs	Derivatives of reactive oxygen metabolites
NPBI	Non-protein-bound iron
AOPP	Advanced oxidation protein products
BAP	Biological antioxidant potential
BALF	Bronchoalveolar lavage fluid
EDTA	Ethylenediaminetetraacetic acid
TNCC	Total nucleated cell count
CV	Coefficient of variation
HPLC	High-performance liquid chromatography
PUFAs	Polyunsaturated fatty acids
IL	Interleukin
COPD	Chronic obstructive pulmonary disease
ARDS	Acute Respiratory Distress Syndrome
IPF	Idiopathic Pulmonary Fibrosis

## References

[1] Couëtil L.L., Cardwell J.M., Gerber V., Lavoie J.P., Léguillette R., Richard E.A. (2016). Inflammatory Airway Disease of Horses—Revised Consensus Statement. J. Vet. Intern. Med..

[2] Fairbairn S.M., Page C.P., Lees P., Cunningham F.M. (1993). Early Neutrophil but Not Eosinophil or Platelet Recruitment to the Lungs of Allergic Horses Following Antigen Exposure. Clin. Exp. Allergy.

[3] Paone G., Conti V., Vestri A., Leone A., Puglisi G., Benassi F., Brunetti G., Schmid G., Cammarella I., Terzano C. (2011). Analysis of Sputum Markers in the Evaluation of Lung Inflammation and Functional Impairment in Symptomatic Smokers and COPD Patients. Dis. Markers.

[4] Fatani S.H. (2014). Biomarkers of Oxidative Stress in Acute and Chronic Bronchial Asthma. J. Asthma.

[5] Kirkham P., Rahman I. (2006). Oxidative Stress in Asthma and COPD: Antioxidants as a Therapeutic Strategy. Pharmacol. Ther..

[6] Reuter S., Gupta S.C., Chaturvedi M.M., Aggarwal B.B. (2010). Oxidative Stress, Inflammation, and Cancer: How Are They Linked?. Free Radic. Biol. Med..

[7] Nakamoto K., Watanabe M., Saito M., Kasuga K., Miyaoka C., Yoshida Y., Kobayashi F., Nunokawa H., Aso J., Nakamoto Y. (2024). Serum Derivatives–Reactive Oxygen Metabolite Levels as a Marker of Clinical Conditions in Patients with Bronchial Asthma, COPD, or Asthma–COPD Overlap: A Prospective Study. J. Clin. Med..

[8] Kotsiou O.S., Tourlakopoulos K., Kontopoulou L., Mavrovounis G., Pantazopoulos I., Kirgou P., Zarogiannis S.G., Daniil Z., Gourgoulianis K.I. (2023). D-ROMs and PAT Tests Reveal a High Level of Oxidative Stress in Patients with Severe Well-Controlled Asthma, and D-ROMs Are Positively Correlated with R20 Values That Indicate Approximate Central Airway Resistance. J. Pers. Med..

[9] Hurrell B.P., Sakano Y., Shen S., Helou D.G., Li M., Shafiei-Jahani P., Kazemi M.H., Sakano K., Li X., Quach C. (2024). Iron Controls the Development of Airway Hyperreactivity by Regulating ILC2 Metabolism and Effector Function. Sci. Transl. Med..

[10] Patel M., Ramavataram D.V.S.S. (2012). Non Transferrin Bound Iron: Nature, Manifestations and Analytical Approaches for Estimation. Indian. J. Clin. Biochem..

[11] Ali M.K., Kim R.Y., Karim R., Mayall J.R., Martin K.L., Shahandeh A., Abbasian F., Starkey M.R., Loustaud-Ratti V., Johnstone D. (2017). Role of Iron in the Pathogenesis of Respiratory Disease. Int. J. Biochem. Cell. Biol..

[12] Wang Z., He Y., Li Q., Zhao Y., Zhang G., Luo Z. (2023). Dysregulation of Iron Homeostasis in Airways Associated with Persistent Preschool Wheezing. Respir. Res..

[13] Ammar M., Bahloul N., Amri O., Omri R., Ghozzi H., Kammoun S., Zeghal K., Ben Mahmoud L. (2022). Oxidative Stress in Patients with Asthma and Its Relation to Uncontrolled Asthma. J. Clin. Lab. Anal..

[14] Nadeem A., Siddiqui N., Alharbi N.O., Alharbi M.M. (2014). Airway and Systemic Oxidant-Antioxidant Dysregulation in Asthma: A Possible Scenario of Oxidants Spill over from Lung into Blood. Pulm. Pharmacol. Ther..

[15] Bullone M., Lavoie J.P. (2017). The Contribution of Oxidative Stress and Inflamm-Aging in Human and Equine Asthma. Int. J. Mol. Sci..

[16] Deaton C.M., Marlin D.J., Deaton L., Smith N.C., Harris P.A., Schroter R.C., Kelly F.J. (2006). Comparison of the Antioxidant Status in Tracheal and Bronchoalveolar Epithelial Lining Fluids in Recurrent Airway Obstruction. Equine Vet. J..

[17] Kirschvink N., Art T., de Moffarts B., Smith N., Marlin D., Roberts C., Lekeux P. (2002). Relationship between Markers of Blood Oxidant Status and Physiological Variables in Healthy and Heaves-affected Horses after Exercise. Equine Vet. J..

[18] Kirschvink N., Smith N., Fiévez L., Bougnet V., Art T., Degand G., Marlin D., Robertson C., Génicot B., Lindsey P. (2002). Effect of Chronic Airway Inflammation and Exercise on Pulmonary and Systemic Antioxidant Status of Healthy and Heaves-affected Horses. Equine Vet. J..

[19] Kramarič P., Pavlica Z., Koklič T., Nemec A., Eržen N.K., Šentjurc M. (2005). Membrane Switch Hypothesis. 2. Domain Structure of Phagocytes in Horses with Recurrent Airway Obstruction. J. Chem. Inf. Model..

[20] Tan R.H.H., Thatcher C.D., Buechner-Maxwell V., Christmann U., Crisman M.V., Werre S.R. (2010). Measurement of Ascorbic Acid Concentration and Glutathione Peroxidase Activity in Biological Samples Collected from Horses with Recurrent Airway Obstruction. Am. J. Vet. Res..

[21] Jochmans-Lemoine A., Picotte K., Beauchamp G., Vargas A., Lavoie J. (2020). Effects of a Propriety Oiled Mixed Hay Feeding System on Lung Function, Neutrophilic Airway Inflammation and Oxidative Stress in Severe Asthmatic Horses. Equine Vet. J..

[22] Reef V.B., Whittier M., Allam L.G. (2004). Thoracic Ultrasonography. Clin. Tech. Equine Pract..

[23] Hoffman A.M. (1999). Bronchoalveolar Lavage Technique and Cytological Diagnosis of Small Airway Inflammatory Disease. Equine Vet. Educ..

[24] Costantini D. (2016). Oxidative Stress Ecology and the D-ROMs Test: Facts, Misfacts and an Appraisal of a Decade’s Work. Behav. Ecol. Sociobiol..

[25] Benzie I.F.F., Strain J.J. (1996). The Ferric Reducing Ability of Plasma (FRAP) as a Measure of “Antioxidant Power”: The FRAP Assay. Anal. Biochem..

[26] Celi P., Sullivan M., Evans D. (2010). The Stability of the Reactive Oxygen Metabolites (d-ROMs) and Biological Antioxidant Potential (BAP) Tests on Stored Horse Blood. Vet. J..

[27] Valerio G., Lacedonia D., Bracciale P., Valerio F. (2016). Determinants of oxidant load in bronchoalveolar milieu. World J. Res. Rev..

[28] Witko-Sarsat V., Friedlander M., Capeillère-Blandin C., Nguyen-Khoa T., Nguyen A.T., Zingraff J., Jungers P., Descamps-Latscha B. (1996). Advanced Oxidation Protein Products as a Novel Marker of Oxidative Stress in Uremia. Kidney Int..

[29] Kime R., Gibson A., Yong W., Hider R., Powers H. (1996). Chromatographic Method for the Determination of Non-Transferrin-Bound Iron Suitable for Use on the Plasma and Bronchoalveolar Lavage Fluid of Preterm Babies. Clin. Sci..

[30] Silva A.M.N., Rangel M. (2022). The (Bio)Chemistry of Non-Transferrin-Bound Iron. Molecules.

[31] Zhang W.Z., Oromendia C., Kikkers S.A., Butler J.J., O’Beirne S., Kim K., O’Neal W.K., Freeman C.M., Christenson S.A., Peters S.P. (2020). Increased Airway Iron Parameters and Risk for Exacerbation in COPD: An Analysis from SPIROMICS. Sci. Rep..

[32] Domej W., Oetll K., Renner W. (2014). Oxidative Stress and Free Radicals in COPD–Implications and Relevance for Treatment. Int. J. Chronic Obstr. Pulm. Dis..

[33] Srinivasamurthy S.K., Mittal P., Goyal A., Ballal S., Maharana L., Goyal K., Rana M., Ali H., Oliver B.G.G., Paudel K.R. (2025). Ferroptosis and Iron Homeostasis in Chronic Obstructive Pulmonary Disease: Therapeutic Opportunities of Iron Chelators. J. Trace Elem. Med. Biol..

[34] Niedzwiedz A., Jaworski Z. (2014). Oxidant-Antioxidant Status in the Blood of Horses with Symptomatic Recurrent Airway Obstruction (RAO). J. Vet. Intern. Med..

[35] Art T., Kirschvink N., Smith N., Lekeux P. (1999). Indices of Oxidative Stress in Blood and Pulmonary Epithelium Lining Fluid in Horses Suffering from Recurrent Airway Obstruction. Equine Vet. J..

[36] Tavilani H., Nadi E., Karimi J., Goodarzi M.T. (2012). Oxidative Stress in COPD Patients, Smokers, and Non-Smokers. Respir. Care.

[37] ben Anes A., Fetoui H., Bchir S., ben Nasr H., Chahdoura H., Chabchoub E., Yacoub S., Garrouch A., Benzarti M., Tabka Z. (2014). Increased Oxidative Stress and Altered Levels of Nitric Oxide and Peroxynitrite in Tunisian Patients with Chronic Obstructive Pulmonary Disease: Correlation with Disease Severity and Airflow Obstruction. Biol. Trace Elem. Res..

[38] Stanojkovic I., Kotur-Stevuljevic J., Milenkovic B., Spasic S., Vujic T., Stefanovic A., llic A., Ivanisevic J. (2011). Pulmonary Function, Oxidative Stress and Inflammatory Markers in Severe COPD Exacerbation. Respir. Med..

[39] Nadeem A., Raj H.G., Chhabra S.K. (2005). Increased Oxidative Stress and Altered Levels of Antioxidants in Chronic Obstructive Pulmonary Disease. Inflammation.

[40] Taniguchi A., Tsuge M., Miyahara N., Tsukahara H. (2021). Reactive Oxygen Species and Antioxidative Defense in Chronic Obstructive Pulmonary Disease. Antioxidants.

[41] Hansen S., Otten N.D., Ceron J.J., González-Arostegui L.G., Peres-Rubio C. (2025). Redox Biomarker Variations With Severity of Asthma in Horses Across Different Sample Types. J. Vet. Intern. Med..

[42] Brkljača Bottegaro N., Gotić J., Šuran J., Brozić D., Klobučar K., Bojanić K., Vrbanac Z. (2018). Effect of Prolonged Submaximal Exercise on Serum Oxidative Stress Biomarkers (d-ROMs, MDA, BAP) and Oxidative Stress Index in Endurance Horses. BMC Vet. Res..

[43] Sgorbini M., Bonelli F., Rota A., Marmorini P., Biagi G., Corazza M., Pasquini A. (2015). Maternal and Neonatal Evaluation of Derivated Reactive Oxygen Metabolites (d-ROMs) and Biological Antioxidant Potential in the Horse. Theriogenology.

[44] Shono S., Gin A., Minowa F., Okubo K., Mochizuki M. (2020). The Oxidative Stress Markers of Horses—The Comparison with Other Animals and the Influence of Exercise and Disease. Animals.

[45] Kawami M., Shimonakamura T., Yumoto R., Takano M. (2018). Transport of AOPP-Albumin into Human Alveolar Epithelial A549 Cell. J. Pharm. Pharm. Sci..

[46] Elkabany Z.A., El-Farrash R.A., Shinkar D.M., Ismail E.A., Nada A.S., Farag A.S., Elsayed M.A., Salama D.H., Macken E.L., Gaballah S.A. (2019). Oxidative stress markers in neonatal respiratory distress syndrome: Advanced oxidation protein products and 8-hydroxy-2-deoxyguanosine in relation to disease severity. Pediatr. Res..

[47] Wang M., Ren R., Xu Y., Wang T., Liang X., Li S. (2024). Oxidative stress in the alveolar lavage fluid of children with Mycoplasma pneumonae pneumonia. Pediatr. Pulmonol..

[48] Liguori I., Russo G., Curcio F., Bulli G., Aran L., Della-Morte D., Gargiulo G., Testa G., Cacciatore F., Bonaduce F. (2018). Oxidative stress, aging and diseases. Clin. Interv. Aging.

[49] Shin N.-R., Kwon H.-J., Ko J.-W., Kim J.-S., Lee I.-C., Kim J.-C., Kim S.-H., Shin I.-S. (2019). S-Allyl Cysteine Reduces Eosinophilic Airway Inflammation and Mucus Overproduction on Ovalbumin-Induced Allergic Asthma Model. Int. Immunopharmacol..

[50] Saastamoinen M., Särkijärvi S., Hyyppä S. (2019). Garlic (Allium Sativum) Supplementation Improves Respiratory Health but Has Increased Risk of Lower Hematologic Values in Horses. Animals.

[51] Xu X., Huang M., Duan X., Liu H., Zhang W., Li D. (2022). Compound Glycyrrhiza Oral Solution Alleviates Oxidative Stress and Inflammation by Regulating SRC/MAPK Pathway in Chronic Obstructive Pulmonary Disease. Immunopharmacol. Immunotoxicol..

[52] Tajvidi E., Nahavandizadeh N., Pournaderi M., Pourrashid A.Z., Bossaghzadeh F., Khoshnood Z. (2021). Study the Antioxidant Effects of Blue-Green Algae Spirulina Extract on ROS and MDA Production in Human Lung Cancer Cells. Biochem. Biophys. Rep..

[53] Nogradi N., Couetil L.L., Messick J., Stochelski M.A., Burgess J.R. (2015). Omega-3 Fatty Acid Supplementation Provides an Additional Benefit to a Low-Dust Diet in the Management of Horses with Chronic Lower Airway Inflammatory Disease. J. Vet. Intern. Med..

[54] Stucchi L., Lo Feudo C.M., Stancari G., Conturba B., Ferrucci F. (2022). Effect of the Administration of a Nutraceutical Supplement in Racehorses with Lower Airway Inflammation. Animals.

[55] Hecker L. (2017). Mechanisms and consequences of oxidative stress in lung disease: Therapeutic implications for an aging populace. Am. J. Physiol. Lung Cell. Mol. Physiol..

